# Systems Biology and Experimental Model Systems of Cancer

**DOI:** 10.3390/jpm10040180

**Published:** 2020-10-19

**Authors:** Gizem Damla Yalcin, Nurseda Danisik, Rana Can Baygin, Ahmet Acar

**Affiliations:** Department of Biological Sciences, Middle East Technical University, Universiteler Mah. Dumlupınar Bulvarı 1, Çankaya, Ankara 06800, Turkey; gizemdyalcin@gmail.com (G.D.Y.); nurseda.danisik@metu.edu.tr (N.D.); rana.baygin@metu.edu.tr (R.C.B.)

**Keywords:** cancer systems biology, experimental model systems, next-generation sequencing, single-cell sequencing, patient-derived xenografts, patient-derived organoids

## Abstract

Over the past decade, we have witnessed an increasing number of large-scale studies that have provided multi-omics data by high-throughput sequencing approaches. This has particularly helped with identifying key (epi)genetic alterations in cancers. Importantly, aberrations that lead to the activation of signaling networks through the disruption of normal cellular homeostasis is seen both in cancer cells and also in the neighboring tumor microenvironment. Cancer systems biology approaches have enabled the efficient integration of experimental data with computational algorithms and the implementation of actionable targeted therapies, as the exceptions, for the treatment of cancer. Comprehensive multi-omics data obtained through the sequencing of tumor samples and experimental model systems will be important in implementing novel cancer systems biology approaches and increasing their efficacy for tailoring novel personalized treatment modalities in cancer. In this review, we discuss emerging cancer systems biology approaches based on multi-omics data derived from bulk and single-cell genomics studies in addition to existing experimental model systems that play a critical role in understanding (epi)genetic heterogeneity and therapy resistance in cancer.

## 1. Introduction to Cancer Systems Biology

**Figure 1:**
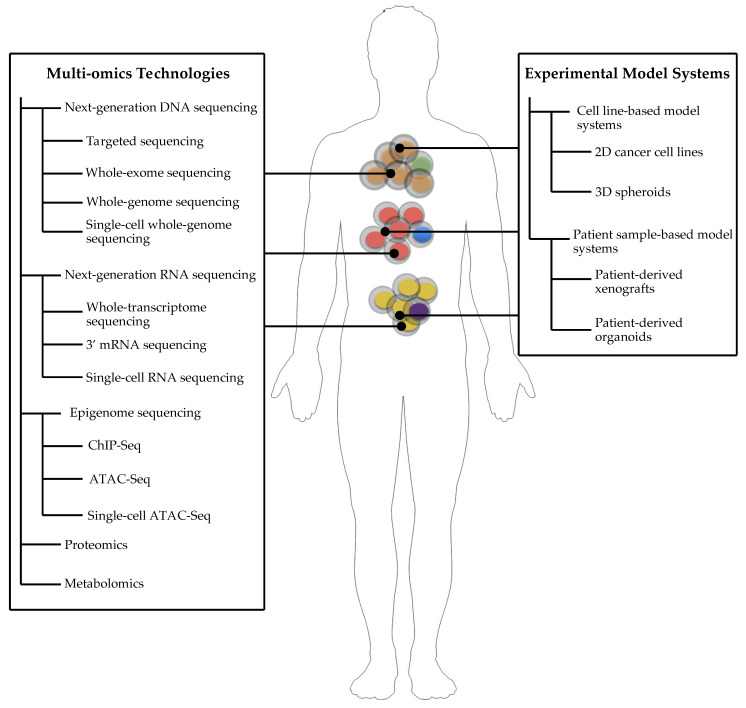
Comprehensive picture of systems biology approaches and experimental model systems constituting the core components of the biology of cancer.

Cancer is an extremely complex disease with heterotypic interactions between cancer cells and neighboring stromal cells that support the proliferation, invasion, and the metastatic cascade of tumor cells [1,2]. Recently, multi-omics approaches empowered by next-generation technologies have enabled genomic characterization and evolutionary histories of both primary and metastatic cancer progression [3,4,5,6]. These technologies that shed light on the genome, transcriptome, metabolome, and proteome of cancer cells corroborate our understanding about systems biology-level approaches in cancer (Figure 1) [7]. Considering the challenges to unify high-throughput data obtained from multi-omics studies, system biology applications in cancer hold a key role to tackle this very problem. For example, cancer as a disease of numerous distinct cell types requires taking into consideration the combination of data derived from these different cell types together with the integration of various layers of genetic and non-genetic data that are forming the cellular systems. Thus, cancer systems biology can simplify the analysis of multi-layer data and offer effective and fast solutions for the development of novel drug technologies and the identification of predictive biomarkers in cancer therapies. Cancer systems biology is an emerging field with accumulating data obtained through network-driven and interdisciplinary science that ultimately aims to tailor better-personalized treatment modalities for patients based on their genetic and non-genetic profiles [8].

The heterogeneous nature of cancers led to studies mapping the (epi)genomic alterations [9,10,11] both in primary [6,12] and metastatic cancers [5]. Through the high-throughput data obtained from cancer patients, it is now possible to combine this information and assess the genotype-to-phenotype link to further characterize the disease onset and clinical outcome. The combination of information derived from the genomic architecture and various gene networks from a single or a group of cells not only determines the fate of these cells during development but also a progression to cancer occurs as a result of the deregulation of these interactions. For example, while the regulation of Notch and Wnt signaling pathways are fine-tuned by each other in normal homeostasis [13], their aberrant expression and deregulation are commonly seen in cancers [14,15]. Therefore, understanding the genetic and epigenetic changes that cause persistent signaling activations and disrupting normal cellular homeostasis is still one of the biggest challenges to address in cancer systems biology.

## 2. Cancer Systems Biology for Precision Medicine

The vast majority of efforts focus on bridging the “big data” obtained from various multi-omics studies to new computational algorithms to ultimately offer more effective personalized cancer therapies. Despite the advancements in cancer therapy through systems biology approaches, treatment resistance is arguably one of the biggest challenges for better-personalized cancer treatments [16,17]. This is mainly due to the fact that cancer follows distinct evolutionary trajectories in patients compared to their genomic landscapes, not only during the initiation and metastasis cascade of cancer cells but also in response to the treatment in cancer therapies [18,19]. For this reason, the accurate identification of subclonal drivers holds great importance for the timing of the subclonal expansion and its diversity in cancer therapies [20]. This sophisticated subclonal identification tool, empowered by machine learning and population genetics, will potentially lead to developing more comprehensive computational methods by integrating with network-driven approaches for cancer systems biology in the future.

With the rapid developments in next-generation sequencing (NGS) technologies, previous microarray studies have been gradually replaced by massively parallel deep sequencing techniques such as whole-genome, whole-exome, targeted-panel, and RNA sequencing [21]. Initially, the microarray platforms have proved to be a very useful tool for genome-wide association studies (GWAS) in cancer systems biology; however, they demonstrated limitations such as covering only a the small fraction of the genome and failure to take into account more than common genetic risk factors [22]. Later, individual research groups started to apply NGS technologies to identify somatic alterations (single-nucleotide variations, copy-number alterations, structural variations) in cancer driver genes and to determine gene expression changes and open chromatin formations both in coding and non-coding regions of the genome [23,24,25,26,27,28]. Then, individual studies were followed by larger multigroup projects [4,5,6]. One of the remarkable efforts is The International Cancer Genome Consortium/The Cancer Genome Atlas (ICGC/TCGA) Pan-Cancer Analysis of Whole Genomes (PWAG) project, comprising a working group of 700 scientists, which recently reported their findings from 2600 whole-genome samples [4,29,30,31]. In addition to these studies that deciphered the evolutionary trajectories of tumors prospectively, recent technologies have also allowed the monitoring of clonal dynamics using “cellular barcodes” integrated into experimental model systems to map the tumor evolution at the single-cell resolution [32,33].

In addition, investigating cancer genomes at the single-cell resolution has taken a big step forward in the past few years [3]. Initial studies focused on the understanding of the transcriptome of single cells in a plate-based system wherein cells were required to be sorted individually, and thus the system lacked high-throughput capacity [34]. However, recent advances, especially with the use of droplet-based systems, have advanced our understanding about single-cell genomics through an increased capacity to profile thousands of single cells at the same time (single-cell RNA sequencing, scRNA-seq) [35,36]. The scRNA-seq technology provided a high-resolution picture not only of cellular states in developmental biology [37] but also in cancer biology where intratumoral heterogeneity and tumor cell plasticity are highly prevalent [38]. Furthermore, the information obtained from the analysis of single-cell WGS (scWGS) has proved to be informative for understanding intratumor heterogeneity and the evolutionary history of thousands of single cells comprising the bulk tumor population [39,40]. Recently, the high-throughput capacity for scWGS has improved significantly, and clonal/subclonal alterations at the single-cell resolution were reported in thousands of cells [41]. To capture the epigenetic changes at the single-cell level, novel methods to map the single-cell epigenome have also been reported. For example, single-nuclei chromatin accessibility assays (ATAC-seq) inferring the chromatin open or closed states in single cells [42,43]. Lastly, the rapid developments in the single-cell biology have also resulted in novel methods such as parallel sequencing of single-cell genomes and transcriptomes [44] and joint profiling of single-cell chromatin accessibility and gene expression [45]. Various online databases containing cancer systems biology tools to document molecular profiles of cancer types are available and offered for the use of the cancer research community (Table 1). Importantly, various multi-omics data obtained using high-throughput sequencing methods enables the integration of these data into experimental model systems for the identification of the actionable targets in cancer. As such, these molecular data integrated with systems biology applications, for the function of transcriptional and proteomics networks, provide effective solutions for the treatment of cancer. Given that cancer is a systems biology disease, integration of the cellular information with the help of computational and mathematical modeling highlights the need to develop more advanced and sophisticated systems biology applications in cancer. This considerable challenge has especially become evident with a rapid increase in the accumulation of sequencing data over the past decade. Hence, to address this very challenge, systems biology approaches are timely positioned to offer novel solutions to better understand the underlying mechanisms of drug resistance and the identification of biomarkers that can predict the disease outcome and response to targeted therapies. Overall, integrating cellular networks with cancer (epi)genomes in both single and bulk cell populations has paved a way to advance our understanding for developing systems biology approaches for precision therapy to advance clinical decisions for patient benefits.

## 3. Experimental Model Systems of Cancer

Although cancer mortality rates are gradually diminishing, it is still one of the deadliest diseases in the world [65]. To develop more effective therapeutic solutions, cancer cell lines, 3D spheroids, in vivo patient-derived xenografts (PDXs), and ex vivo patient-derived organoids (PDOs) have been studied by various groups [66,67,68]. Due to the advances in the development of experimental model systems, there has been remarkable progress in understanding the underlying mechanisms of initiation, progression, and the metastatic cascade of cancer cells [69]. In addition to the advantages of each model system, traditional model systems have failed to recapitulate the response to drugs that are observed in the clinic. For instance, targeted therapies and chemotherapeutic agents that work well in preclinical model systems fail to proceed into clinical trials since specific model systems were unable to recapitulate the disease progression [70]. Therefore, in this section of the review, we sought to discuss current preclinical model systems used in cancer research and their role in predicting how cancer will progress and respond to the therapy when these model systems are integrated with system biology approaches.

## 4. Cell Line-Based Model Systems

Since the first human cancer cell line was established in 1951, 2D monolayer systems have provided major advantages in the understanding of tumor biology and cancer therapy [71]. Over the decades, 2D monolayer systems offered several advantages such as being easy to expand and hence allowing long-term culture times, being manipulated by gene insertions and deletions, and requiring inexpensive material for culturing [72]. On the other hand, this platform has many drawbacks, mainly its inability to mimic the 3D nature of tumor growth. The inadequacies of the 2D monolayer systems also include a lack of cell-to-extracellular matrix (ECM) contact that has been reported as responsible for the accurate detection of cell viability/death, drug metabolism, and expression of certain genes and protein in tumors [73]. Another major limitation of 2D monolayer systems is their inaccurate utility of oxygen and nutrients when compared to 3D culture systems that have proven to be more successful in mimicking real tumor masses [74]. Collectively, 2D monolayer systems have played a major role in understanding and designing cancer therapies for systems biology approaches; however, due to their insufficiency to predict real tumor outcomes in patients, more suitable model systems such as 3D culture systems have been developed.

The first 3D culture was performed using a soft agar solution by Hamburger and Salmon in 1977 [75]. Since that time, several 3D culture methods have been documented. Depending on the material used, the 3D culture systems can be divided into three categories: (i) cultured onto non-adherent plates, (ii) embedded into matrigel-like substances, and (iii) seeded into scaffold-based systems. The general approach for 3D culture systems is based on the formation of a spheroid structure in which cancer cells can form various layers. The 3D nature of spheroids has been demonstrated as a successful system in mimicking the features of the solid tumor mass [73]. Three-dimensional spheroids can also mimic tissue-specific functional characteristics in developmental processes. For example, cardiomyocyte spheroids can exhibit heart-like rhythms, and hepatocyte spheroids exhibit biochemical functions of the liver [76,77]. Three-dimensional culture systems have also been shown to mimic in vivo-like microenvironments via the establishment of complex cell-to-cell and cell-to-ECM communications. These interactions result in cellular signal transduction events similar to tumor tissues that can mediate their cell shape and proliferation [78]. In addition, drug response assays in 3D culture systems were shown to resemble in vivo studies more than 2D culture systems in terms of their success rates in preclinical studies [79,80]. In another study, sensitivities of the same cell line against different chemotherapeutic agents were reported as different in 2D vs. 3D culture systems [81]. For instance, in this study, HCT-116 cells grown in both 2D and 3D model systems and their sensitivities against four commonly used anticancer agents (melphalan, 5-Fluorouracil, oxaliplatin, and irinotecan) were tested. The efficacy of these inhibitors was higher in the 2D than the 3D culture system, suggesting that phenotypic differences and distinct cell-to-cell interactions between these model systems might be responsible for observing the differences in drug sensitivities.

## 5. Patient Sample-Based Model Systems

Patient-derived xenografts (PDXs) are preclinical models established by directly transplanting patient-derived tumor specimens into immunodeficient mice [82]. PDXs have been accepted as promising preclinical model systems that successfully mimic the testing of anticancer drugs [66]. This system provides several advantages, such as the preservation of tumor heterogeneity, molecular subtypes and the clinicopathological features of the tumors obtained from patients [83]. In addition, PDXs have been shown to successfully predict the drug response in the preclinical setting to test the effectiveness of therapeutic agents [84]. While PDXs offer several advantages as a preclinical model system, an increasing body of evidence suggests there are limitations [85]. Firstly, a significant proportion of tumor samples engrafted in mice may not successfully grow due to the host mouse environment causing a bottleneck. Secondly, engraftment times can be long so that the maintenance costs associated with each PDX prove prohibitive. Thirdly, there is still no standardized method for choosing the type of mouse or engraftment technique specific for each cancer type, which raises the possibility of obtaining non-reproducible results between different studies. Studies that overcome these limitations have shed light on the mechanisms of acquired drug resistance, especially in metastatic colorectal cancer (mCRC). For instance, a series of seminal studies published by the Bertotti Lab has demonstrated the use of a large PDX biobank to investigate the underlying mechanisms of drug resistance in mCRC [86,87,88,89]. Importantly, one of these studies played a critical role in assessing the genomic landscape of anti-EGFR antibody blockage in PDXs and functional consequences linked to clinical data in cancer patients [87]. Thus, PDXs have paved a way to develop a platform for the systematic analysis and evaluation of cancer therapies.

Patient-derived tumor organoids (PDOs) are ex vivo three-dimensional structures of tumors obtained from cancer patients and grown in the presence of an extracellular matrix [90]. Accumulating evidence suggests that PDOs can successfully predict the drug response in cancer patients in the clinic in addition to preserving the genetic and transcriptomic heterogeneity of the original tumor [67]. In addition, studies focused on comparing the histopathological features of tumors with PDOs revealed that the PDOs maintain similar morphological characteristics as the original tumor [90,91]. Importantly, PDOs also mimic the genomic and transcriptomic features of the tumors that they have derived from even after long ex vivo culture times [91,92,93]. To date, PDOs have been established from different cancer types including colorectal [93], gastrointestinal [91], pancreatic [94], prostate [95], bladder [96], breast [97], glioblastoma [98], and ovarian [99]. Three-dimensional cultures of PDOs that predict the outcome of drug treatment in cancer patients can be considered an important milestone for personalized medicine for the benefits of cancer patient [100]. When PDOs are established from individual patients in a short time, they can provide a window of opportunity to test therapeutic agents in parallel to the clinic, and thus the outcome of drug testing in the laboratory can prove informative for the decision making of treatment for patients.

Amongst the key studies about PDOs, van de Wetering et al. (2015) is the first study that reported a well-established and characterized PDO biobank from 20 primary CRC patients [93]. In this study, whole-exome sequencing (WES) and the RNA sequencing of samples resulted in preserved genetic heterogeneity and molecular cancer subtypes both in the primary tumor tissue and PDOs. In addition, the genetic heterogeneity of the primary tumor was mostly preserved during the establishment and long culture times of organoids in ex vivo. The histopathological assessment of samples suggested a very high similarity in terms of the phenotypic heterogeneity between PDOs and the parental tumor. In this important study, PDOs were treated with 58 chemotherapeutic agents, and those with TP53 loss of function mutation were resistant to MDM2 inhibitors and as a consequence acquired RAS mutations and therefore decreased sensitivity to an EGFR inhibitor. Importantly, in this study, colon tumor organoids carrying the RNF43 mutation were dramatically sensitive to Wnt inhibitors.

In another significant study, PDOs were examined for the first time to investigate whether PDOs as a preclinical model could predict the drug response seen in the gastrointestinal cancer patients in the clinic [91]. In this study, a living organoid biobank was established from metastatic gastrointestinal cancer patients who were previously recruited for phase I or II clinical trials. According to the phenotypic and genotypic profiling of organoid and patient tumor samples, both of them exhibited highly similar profiles. Then, this led the authors to assess drug responses of PDOs in the laboratory setting in parallel to the clinic. High-throughput drug screening of PDOs with Food and Drug Administration (FDA)-approved drugs was shown to be successful with a positive predictive value (predicting that a certain drug worked) of 88% and a negative predictive value (predicting that a certain drug did not work) of 100%. This suggests a promising forecasting potential for PDOs in terms of the treatment response.

## 6. Conclusions

Extensive (epi)genetic heterogeneity in cancer has been demonstrated in several studies. As a result of the aberrantly activated and sustained complex signaling networks both in cancer cells and neighboring tumor microenvironment, examples of the hallmarks of cancer were presented. To address genomic aberrations and signaling network complexity, there has been a growing need to develop more sophisticated approaches for cancer systems biology. Cancer systems biology can deliver solutions for the better understanding of intratumor heterogeneity and therapeutic opportunities. Specifically, improved cancer systems biology approaches integrated not only with multi-omics data from tumors but also with comprehensive patient-derived experimental model systems can guide clinicians for their decision-making to offer better therapeutic solutions with an ultimate aim to overcome treatment failure in cancer.

## Figures and Tables

**Table 1:** A collection of databases.

Name	Description	Website	Reference
CaSNP	CaSNP performs quantitative analysis of copy number variation from SNP arrays in multiple cancer types	https://bioinformaticshome.com/tools/cnv/descriptions/CaSNP.html	[46]
OncoLand	OncoLand provides oncology data access in sample and gene directions.	https://omicsoftdocs.github.io/ArraySuiteDoc/tutorials/OncoLand/Introduction/	[47]
AGCOH	The Atlas of Genetics, Cytogenetics in Oncology and Hematology perform comprehensive genomic characterization and analysis of multiple cancer types	http://atlasgeneticsoncology.org/BackpageAbout.html	[48]
PCWAG	PCWAG—Pan-cancer Analysis of Whole Genomes provides common patterns of mutations from more than 2600 cancer whole genomes	http://dcc.icgc.org/pcawg	[4]
ChiTaRS	ChiTaRS contains chimeric transcripts and RNA-Seq data	http://chitars.bioinfo.cnio.es/	[49]
CanSAR	CanSAR provides information about translational research and drug discovery knowledgebase	https://cansarblack.icr.ac.uk/	[50]
OncoDB.HCC	Oncogenomics Database of Hepatocellular Carcinoma provides genomic, transcriptomic, and proteomic data	http://oncodb.hcc.ibms.sinica.edu.tw/index.htm	[51]
COSMIC	COSMIC performs a comprehensive database of somatic mutation in multiple cancer types	https://cancer.sanger.ac.uk/cosmic	[52]
canEvolve	canEvolve is a comprehensive database including genes, miRNA, and protein expression profiles; copy number changes for a variety of cancer types and protein–protein interactions	http://www.canevolve.org/AnalysisResults/AnalysisResults.html	[53]
CancerPPD	CancerPPD provides information about anticancer peptides and proteins in multiple cancer types	http://crdd.osdd.net/raghava/cancerppd/	[54]
PED	The Pancreatic Expression Database performs a comprehensive meta-analysis of pancreatic cancer	http://www.pancreasexpression.org/	[55]
CGP	Cancer Genome Project provides genotype and copy number changes information in tumors	https://www.sanger.ac.uk/group/cancer-genome-project	[56]
MethyCancer	MethyCancer provides information about DNA methylation and gene expression in a variety of cancer types	http://methycancer.psych.ac.cn/	[57]
CPTAC	Clinical Proteomic Tumor Analysis Consortium is a database containing an integration of genomic and proteomic data	https://proteomics.cancer.gov/	[58]
intOGen	Integrative Onco Genomics performs comprehensive genomic data of multiple cancer types	https://www.intogen.org/search	[59]
ArrayExpress	ArrayExpress focuses on microarray gene expression data	https://www.ebi.ac.uk/arrayexpress/	[60]
DriverDBv3	DriverDBv3 is a database of cancer omics	http://driverdb.tms.cmu.edu.tw/	[61]
PCDB	The Pancreatic Cancer Database provides genetic information in pancreatic cancer	http://www.pancreaticcancerdatabase.org	[62]
CancerDR	CancerDR contains anticancer drugs and their effectiveness against a variety of cell lines	http://crdd.osdd.net/raghava/cancerdr/	[63]
Platinum	Platinum provides knowledge about missense mutations on ligand–proteome interactions	http://biosig.unimelb.edu.au/platinum/	[64]
